# Personality-Targeted Interventions for Substance Use and Misuse

**DOI:** 10.1007/s40429-016-0127-6

**Published:** 2016-11-04

**Authors:** Patricia J. Conrod

**Affiliations:** Department of Psychiatry, Université de Montréal, Centre de Recherche, CHU Ste-Justine, Chercheur Boursier Senior, FRSQ, 3175 Côte-Ste-Catherine, Montréal, QC H3T 1C5 Canada

**Keywords:** Prevention, Substance misuse, Adolescents, Personality, Selective interventions

## Abstract

**Purpose of Review:**

Personality factors have been implicated in risk for substance use disorders through longitudinal and neurobiologic studies for over four decades. Only recently, however, have targeted interventions been developed to assist individuals with personality risk factors for substance use disorders manage their risk. This article reviews current practices in personality-targeted interventions and the eight randomised trials examining the efficacy of such approaches with respect to reducing and preventing substance use and misuse.

**Recent Findings:**

Results indicate a moderate mean effect size for personality-targeted approaches across several different substance use outcomes and intervention settings and formats.

**Conclusions:**

Personality-targeted interventions offer several advantages over traditional substance use interventions, particularly when attempting to prevent development of problems in high-risk individuals or when addressing concurrent mental health problems in brief interventions.

## Substance Misuse in North America

Despite having made great strides in reducing adolescent binge-drinking rates, illicit substance use remains significantly above national targets for health promotion and disease prevention in the USA and Canada [1–3]. In 2014, marijuana use and nonmedical use of psychotherapeutics were the most common types of illicit drug use by North American adolescents and there is very little evidence that rates of cannabis use have changed over the past 10 years. More concerning is that rates of adolescent *substance use disorder* remain high and unchanged over this period [3]. These data suggest that there is a need to shift the focus of prevention efforts away from universal approaches to more targeted intervention strategies designed to help those most at risk of transitioning to substance use disorders.

Personality factors have been identified as robust risk factors for substance use disorders and have been shown to mediate the genetic predisposition to substance misuse and predict patterns of psychiatric comorbidity and patterns of substance misuse (see [4•] for review). Inspired by these findings, the personality-targeted approach to substance use prevention and brief intervention offers a novel strategy for intervening on risk factors for substance misuse and offers many advantages over more traditional universal prevention or generic intervention approaches targeting substance use behaviours more directly. The current review provides an overview of current practices and empirical findings on personality-targeted interventions for substance misuse, as well as a brief overview of the literature linking personality factors to substance use risk, which is also addressed in more detail in previous publications [4•, 5•].

### Substance Use Among Young People

While epidemiologic studies in North America and Europe are indicating that rates of underage drinking and binge drinking have been decreasing over the past 10 years, alcohol- and drug-related harms remain alarmingly prevalent among adolescents and young adults. A recent World Health Organization study [6] reported that alcohol use alone accounts for almost 4 % of the global burden of health, with deaths attributed to alcohol greater than those caused by AIDS, tuberculosis or violence. Heavy drinking is very common among undergraduate students: 40–60 % engage in heavy episodic drinking (HED; 5+ drinks/occasion) [7–9]. Recent studies, including one of drinking patterns in 73 countries, show that risky drinking patterns, such as drinking to intoxication and HED, are on the rise among young adults [6, 10, 11]. Heavy alcohol use negatively impacts physical health, psychological well-being and academic performance [12–14]. Such alcohol-related negative consequences occur frequently among undergraduates. For example, a large study of Canadian undergraduates estimated that a full 44 % of students reported one or more indicators of harmful drinking such as experiencing memory loss, suffering an injury, feeling guilty or having other concerns about their drinking [7]. An estimated 18–27 % of undergraduates will experience an alcohol use disorder in their lifetime [15]. In addition, one third of undergraduates report one or more indicators of dependent drinking such as being unable to stop drinking and needing a drink in the morning or hazardous patterns of drinking according to the Alcohol Use Disorders Identification Test [16]. This research suggests that a majority of students are negatively affected by drinking on campus [7], whether it is missing classes due to hangover (19 %), engaging in unplanned sex (14.1 %), driving (7.4 %) or unsafe sex (6.0 %) after drinking or experiencing negative consequences due to other students’ drinking such as being assaulted (10.0 %) or sexually harassed (9.8 %).

There is also a widespread concern about a prescription drug crisis currently facing North America [2]. This concern appears particularly warranted in the case of young people, where 15–25 year olds report the highest rates of prescription drug misuse of any age group [3]. Non-medical prescription drug misuse (NMPDM) is indeed a risk factor for future prescription drug dependence [17] and has been linked to the rise in heroin abuse in the USA [18]. The number of overdose deaths from prescription pain relievers has more than quadrupled since 1999 in the USA [19]. There is a pressing need for effective substance abuse prevention and early intervention, yet very few programmes have been developed to address illicit drug use among youth and even fewer have been evaluated with respect to their efficacy in preventing onset of prescription drug use.

### Personality Factors and Risk for Substance Use and Misuse

Externalising and internalising personality traits are reliably associated with increased susceptibility for substance misuse in youth, with a growing body of evidence suggesting that these traits play a causal role in substance misuse vulnerability. Consequently, these personality profiles have recently become important targets of interventions to reduce such risk. We previously showed that distinct personality traits are related to risk for substance-specific misuse patterns, with impulsivity (IMP) specifically associated with misuse of stimulants (including cocaine and prescription stimulant medications) and sensation seeking (SS) preferentially associated with alcohol and cannabis misuse [20–24]. By contrast, anxiety sensitivity (AS) and hopelessness (HOP) have been shown to be associated with preferential use/misuse of depressant drugs, such as alcohol, sedatives and opioids [23, 25]. These traits also appear to predict different motives for drinking and substance use with SS being consistently associated with enhancement motives for drinking and drug use, AS with coping and conformity motives and HOP with a specific motivation to manage painful emotions and memories [23, 26, 27]. Interestingly, IMP has been shown to be associated with a motivationally undefined pattern of substance use, whereby all drinking and drug use situations (availability) appear to motivate substance use in high-impulsive individuals [23, 26].

These findings have stimulated an interest in understanding the cognitive and motivational factors that distinguish individuals with these personality traits from those without to inform the development of new interventions. With respect to IMP, a very large literature points to a specific deficit in response inhibition, which has been linked to abnormal structural and functional brain profiles that suggest a brain-related impairment in stopping behaviour [28, 29]. This research also suggests that these deficits are directly related to the tendency to drink and try substances in early adolescence and to be more susceptible to more frequent and excessive use following onset (compulsive use).

By contrast, SS has also been shown to be associated with a unique neurocognitive profile that differs from IMP in that it is necessarily dependent on a motivational state, particularly incentive reward or reward anticipation. Sensation seekers, by definition, report being highly motivated by (or sensitive to) the presence or absence of incentive reward. Studies involving self-report, cognitive and neuroimaging measures have shown individuals high in SS to become particularly undercontrolled and reactive when anticipating incentive reward [28, 30]. However, under conditions where behaviour is motivated by avoidance of punishment, for example, they show normal or superior performance on tasks of cognitive and behavioural control [28, 31]. Sensation seekers also appear to be sensitive to incentive reward when it is provided in a pharmacological form. Numerous studies report that SS is associated with heightened reactivity, or sensitivity, to the pharmacological effects of alcohol and stimulants on indices of incentive reward, such as subjective high, cardiac acceleration [32, 33] and increased post-synaptic availability of dopamine in the nucleus accumbens [34].

AS and HOP are associated with alcohol and prescription depressant drug use and misuse (including sedatives and narcotics) [22, 23] through different motivational processes, mainly negative reinforcement or removal of negative emotional states. Both traits are associated with elevated risk for internalising problems in adolescents and adults [31, 35], which are also characterised by a cognitive sensitivity to negative information [31]. AS might also be associated with a more specific sensitivity to threat cues, particularly interoceptive cues [36], but studies investigating whether youth with these pre-morbid risk factors are also characterised by attentional biases to threat and negative information have yielded inconsistent findings [37]. It has yet to be confirmed whether, or not, cognitive or attentional biases are causally implicated in risk for substance misuse in youth with high levels of AS or HOP, but the evidence does demonstrate a direct link between these traits and risky motives for substance use, which directly increase one’s risk for problem drinking (25–27;61).

### Targeted Prevention for Youth Substance Misuse

Reviews of school-based drug prevention programmes conclude that the majority of such interventions are universal drug awareness programmes that are either untested or that have only mild positive or even harmful effects [38, 39, 40]. There has been much more systematic research on effective prevention of hazardous drinking in undergraduates [41, 42]. Recent reviews of the characteristics of effective school-based hazardous drinking prevention programmes conclude that targeting high-risk students and using interactive activities are components essential to maximising efficacy [43, 44]. A set of recommendations from the National Institute on Alcohol Abuse and Alcoholism [45] identified three types of targeted programmes that are most effective (i.e. “Tier I″) in prevention of hazardous drinking among undergraduates: programmes employing a combination of cognitive–behavioural skill training and motivational enhancement; those involving brief motivational enhancement; and those challenging positive alcohol expectancies. Motivational enhancement techniques also show promise in undergraduate drug use prevention [46]. These identified characteristics are all components that can be included in interventions that differentially target personality risk profiles for substance misuse.

### The Substance Use Risk Profile Scale: a Brief Personality Risk Assessment Scale

The Substance Use Risk Profile Scale (SURPS) was developed to assess four personality traits relevant to substance misuse risk: AS, HOP, IMP and SS. The 23-item SURPS was developed and validated by Woicik, Stewart, Pihl and Conrod [23] using factor analysis on a battery of personality and symptom inventories that tap these four personality dimensions and is the only brief personality assessment tool that provides relatively independent measurement of these four personality traits. It is suitable for self-administration by adolescents and adults [23], and the brevity of the scale is highly advantageous in applied research contexts where large numbers of participants are screened simultaneously or complete the scale as part of a larger assessment battery. It has also proven useful in clinical settings where time limitations are significant barriers to using psychometric tests. The SURPS has been translated into French, German, Dutch, Czech, Spanish, Japanese, Sri Lankan, Cantonese, Mandarin, Hebrew and Turkish and has shown good internal consistency, test–retest reliability and concurrent and predictive validity with respect to identifying current and future substance misuse among adolescents and young adults across many different cultural and political contexts (e.g. [23, 47–49]). Importantly, the SURPS has also been shown to have incremental validity over the NEO-FFI scales in predicting drinking problems [23] and prospective validity in predicting substance use outcomes [23, 30, 50, 51], as well as mental health outcomes [30, 37]. These studies also show that the SURPS subscales are specifically predictive of different patterns of psychopathology in theoretically relevant ways [28, 30, 31].

### Personality-Targeted Interventions for the Prevention of Alcohol and Illicit Drug Misuse: the Preventure Programme

The Preventure Programme was designed to target known personality risk factors for substance misuse based on the evidence accumulated thus far on effective interventions for youth alcohol and substance misuse [52]. Unlike universal programmes that tend to universally promote generic coping skills and balance normative attitudes around substance use, this selected personality-targeted approach targets four personality-specific motivational pathways to substance misuse: HOP, AS, IMP and SS.

After selection on personality scales (often using the SURPS), high-risk individuals are invited to participate in brief individual- or group-based intervention sessions that target their dominant personality profile. Interventions are generally two-to-six sessions in duration, with 1 week separating (each-remove) session, each generally 90 min in duration. The interventions are conducted using manuals that (remove which) incorporate psycho-educational, motivational enhancement therapy (MET; [53]) and cognitive–behavioural (CBT; [53]) components and include real-life ‘scenarios’ shared by local youth with similar personality profiles. In the first session, participants are guided in a goal setting exercise, designed to enhance motivation to change behaviour. Psycho-educational strategies are then used to teach participants about the target personality variable and associated problematic coping behaviours like avoidance, interpersonal dependence, aggression, risky behaviours and substance misuse. They are then introduced to the CBT model and guided in breaking down a personal experience according to the physical, cognitive and behavioural components of an emotional response. In the subsequent sessions, participants are encouraged to identify and challenge personality-specific cognitive distortions that lead to problematic behaviours.

The main difference between the personality-targeted approach and other brief intervention strategies (e.g. brief motivational interviewing; [53]) is that each component is introduced and discussed in personality-specific ways. For example, the IMP intervention will discuss drug and alcohol expectancies as they pertain to IMP, as well as promote the development of cognitive behavioural skills that are most relevant to cognitive control and response inhibition, whereas the AS intervention will challenge expectancies related to the positive nature of anxiolytic substances, while also helping high-risk youth learn to challenge their catastrophic reactions to interoceptive cues and reduce avoidance behaviours in response to such cues. The cognitive–behavioural strategies that are used in the personality-targeted approach are closely based on the evidence-based strategies that would be used in CBT interventions for major psychiatric disorders to which each of these personality profiles is most relevant, for example, CBT for depression in the case of HOP (e.g. [54]), CBT for panic disorder in the case of AS (e.g. [55, 56]) or CBT for ADHD in the case of IMP (e.g. [57]).

The one intervention for which very little evidence-based strategies were available at the time of developing the Preventure Programme was the intervention targeting SS. Our research has shown that one of the most important distinctions between SS and IMP is that SS does not appear to be related to risk for the other forms of non-addictive psychopathology. Therefore, sensation seekers tend not to be seen in general psychology or psychiatry clinics: As a consequence, other than interventions for substance use disorders, few CBT intervention strategies have been developed to help sensation seekers moderate the cognitive and behavioural tendencies that contribute to their difficulties in life.

As outlined above, research on the cognitive and neural characteristics of SS consistently indicates a sensitivity to reward, whereby such individuals demonstrate more disinhibition from incentive reward but might also demonstrate abnormal subcortical (e.g. limbic) activation patterns to reward that would also leave an individual feeling less subjective reward from conditions where reward is less immediate or ambiguous [58]. By contrast, high doses of substances appear to be more stimulating for sensation seekers than for non-sensation seekers. These findings have informed the focus of our SS intervention, in which cognitive behavioural exercises are used to help sensation seekers identify situations in which their tendency to ‘chase the fun’ leads to unwanted consequences for them. Youth are guided in discussing how using substances (used-remove) to cope with the need for stimulation can be problematic. They are then assisted in exploring substance-unrelated strategies for managing their need for stimulation or their tendency to become undercontrolled under highly incentive rewarding situations.

The programme does not focus on substance use but rather on risky personality-based ways of coping that may lead to substance misuse or other risky behaviour, such as aggressive thinking, interpersonal dependence and avoidance. Therefore, only one page of the 35-page treatment manual [59] directly refers to substance use as a risky coping strategy, and an additional couple of scenarios might describe substance use by secondary characters mentioned in high-risk scenarios.

### Delivery Format

When applied to the school setting, interventions are only two 90-min group sessions facilitated by a trained facilitator and co-facilitator, with a minimum of 1 week between sessions [60, 61, 62•, 63•]. Youth with similar personality profiles are grouped together to complete personality-specific interventions targeting their most salient personality profile. In more clinically oriented settings, such as psychiatry clinics or special education institutions, where youth might suffer from more severe learning difficulties or psychiatric comorbidity, interventions can be broken down into multiple briefer intervention sessions, which can be delivered in a group or individual format, depending on the individual needs of the client. In addition, a very novel treatment delivery approach [44, 64] recruited participants living with anxiety disorders from the community and simply mailed treatment manuals to their homes. Intervention sessions were conducted individually with the assistance of a coach who is available to participants by telephone or email.

### Developmental and Cultural Adaptations of Personality-Targeted Interventions

Programmes that are sensitive to the developmental needs, cultural values and attitudes of a target group are more effective and reported by adolescents to be more relevant [65, 66]. Therefore, for every new implementation of the Preventure Programme, a preliminary process of developmental and cultural adaptation of intervention materials is recommended. First, it is recommended that the SURPS be translated, back-translated and then evaluated for internal consistency. It is also recommended that the scale be administered to a representative sample of target participants in the new context to confirm that personality factors are indeed related to substance use and misuse in that context. For example, when adapting the programme for youth living in First Nations Communities in Canada, it was not at all clear whether personality factors played a similar role in their substance use, as had been previously demonstrated for youth attending mainstream schools in Canada, as reported in [23]. This research showed that the personality model was highly relevant to substance use in First Nations youth [67]. Similar cultural adaptations of the scale have been published in advance of programme adaptation (e.g. [68, 69]).

Additional validation procedures can include qualitative interviews with high-risk youth identified using the SURPS, such as described by Barrett et al. [70], and procedures that include even more community engagement, such as described by Mushquash et al. [71, 72]. In both adaptations, a mixed-method approach was used in which quantitative surveys such as the Drinking Motives Questionnaire are used to confirm different motivational profiles in high-risk youth and qualitative surveys and interviews with high-risk youth are used to collect detailed information on where drinking and drug use situations occur in young people’s lives and other local interests and pastime activities for youth. Conducting structured qualitative interviews with youth who reported substance use and elevated personality risk is also recommended to help identify local terms used to describe substance-related activities and the physical and emotional states relevant to each personality dimension. For example, when adapting the Preventure Programme to French Canadian youth, it became evident that the term ‘Drinking to get high’ did not have a direct translation in French but rather translated to a more ambiguous term, “Pour le ‘*Feeling*”’. While it was important to maintain the local term for feeling intoxicated, it was also important to add additional material to some of the scenarios to allow for motivational differentiation across drinking situations (e.g. to distinguish drinking for anxiety management vs. for enhancement motives). All this qualitative information is then directly used to create relevant high-risk scenarios that are included in manuals and which are read aloud during intervention sessions to help young people better understand a particular cognitive behavioural process or high-risk situation.

Another important step in some adaptations has been to have local educational and/or psychological professionals review intervention materials and provide detailed feedback on the developmental appropriateness of the content for a particular age group or clinical population. This was particularly relevant when adapting the Preventure Programme for youth in London, UK and Montreal, in which the goal of the study was to test the impact to the programme for younger cohorts than in previous trials in order to demonstrate prevention of substance use *onset*. The recent Montreal and Australian adaptations [51] were also reviewed by experienced editors of children’s literature to be sure that the intervention material, particularly the scenarios, is written in a way that is engaging for readers of that age.

Finally, it is also recommended that new adaptations are first piloted with high-risk youth, who are then asked about their experiences with the intervention. According to one qualitative evaluation of adolescents’ reactions to interventions, they report that, for them, the most important components of the intervention are learning cognitive–behavioural strategies and that such skill development during personality-targeted interventions was key to positive behavioural change [73•]. Importantly, youth-generated information regarding their intervention experiences independently accounted for 12–25 % of the variance in change in alcohol use and mental health symptoms over 12 months. By contrast, very little variance in substance use outcomes could be predicted using investigator-selected quantitative measures of cognitive–behavioural processes, suggesting that mixed-method approaches, particularly those that allow for youth perspectives to be communicated, are extremely important in the adaptation process.

### Training Educational and Health Professionals to Deliver Personality-Targeted Interventions

In a study of effectiveness of the Preventure Programme under real-world conditions, O’Leary-Barrett and her colleagues [74] described a procedure by which educational professionals were trained to implement the programme through a structured training protocol involving a 3-day workshop and two supervised practical sessions in which trainees delivered a two-session intervention to high-risk youth. Preventure trainers offered supervision and standardised feedback using a scale that was developed to evaluate adherence to 12 core treatment components of the personality-targeted intervention programme, such as goal setting and identifying and challenging automatic thoughts [73•]. The Cognitive Therapy Scale—Revised [75] and the Motivational Interviewing Treatment Integrity 3.0 [76] were also used to provide trainees with feedback on the quality of their therapy-specific skills. In this trial and subsequent trials, each trial facilitator must reach sufficient levels of programme delivery before running personality-targeted interventions with trial participants. This procedure is now used rather systematically to disseminate the programme to different communities around the world and has proven to be effective, not only in transferring skills to new clinical teams [74], but also leading to behavioural changes in young people [77], particularly if treatment fidelity is measured during programme implementation [51].

### A Review of the Evidence on Personality-Targeted Interventions for Substance Misuse

Whether it involves the delivery of the full Preventure Programme or personality-specific interventions, the personality-targeted approach has now been evaluated in eight randomised trials, with two additional trials in progress. Published trials are summarised in Table 1 and include standardised effect size estimates for primary outcomes: alcohol use, binge drinking, problem drinking and illicit or prescription drug use. Where possible, results of quantity and frequency outcomes are also included. While this does not include a systematic review and formal meta-analysis, the trials summarised in this table include all published trials of personality-targeted interventions to date. These trials typically target individuals who are considered at high risk of misusing substances prior to the onset, use or problem use of substances. Therefore, many of the trials are conducted with secondary school students who have been invited to participate in interventions because they scored one standard deviation above the mean on one of the SURPS measures. Some such trials also specified drinking onset as an additional eligibility criterion (e.g. [60, 77]). Two trials differ in that they target adults recruited from the community living with mental health problems, such as substance dependence [22] or anxiety disorders [44]. The school-based studies delivered interventions in group format, while the adult studies delivered interventions in an individual format. Finally, studies also vary in terms of duration of follow-up from 4 months to 3 years.

**Table 1:** Summary of eight randomized trials of personality-targeted interventions for substance misuse and standardized effect sizes (Cohen’s *d* equivalent)

Trial	Personality traits targeted	Population targeted	Behavioural outcomes targeted	Effect sizes all reported as Cohen’s *d*
1. Montreal Prescription Drug and Alcohol Dependence Trial [22]	IMP/SS, AS, HOP	Alcohol and/or prescription drug- dependent women Int: *n* = 78 Ctr: *n* = 45	Alcohol use Alcohol QF Dependence symptoms Remission prescription drug use	0.47 (0.10 to 0.84)* 0.02 (−0.35 to 0.39) 0.47 (0.10 to 0.84)* 0.46 (0.10 to 0.83)* 0.58 (0.03 to 1.13)*
2. Canadian Preventure Trial [60]	AS, SS, HOP	HR secondary students (drinkers) Int: *N* = 166 Ctr: *n* = 131	Alcohol use (4 months) Binge drinking (4 months) Drinking problems (4 months)	0.20 (−0.02 to 0.43) 0.37 (0.14 to 0.60)* 0.32 (0.09 to 0.55)*
3. College AS Trial^a^ [78]	AS	College students Int: *n* = 51 Ctr: *n* = 56	Drinking frequency Binge drinking Drinking problems	00 (ns) Not reported 0.37 (−0.02 to 0.75)
4. UK Preventure Trial^b^ [61, 62•, 81]	AS, IMP, HOP, SS	HR secondary students Int: *n* = 190 Ctr: *n* = 157	Alcohol use (6 months) Binge drinking (6 months) Drinking problems (6 months) Drinking problems (2 years) Drug use frequency (2 years) Cannabis use (2 years) Cocaine use (2 years)	0.22 (0.00 to 0.43)* 0.21 (0.00 to 0.42)* 0.35 (0.00 to 0.42)* 0.33 (0.12 to 0.54)* 0.25 (0.10 to 0.40)* 0.16 (0.04 to 0.34)*^d^ 0.80 (0.94 to 1.17)*^d^
5. Dutch Preventure^c^ Trial [77]	AS, IMP, HOP, SS	HR secondary students (drinkers) Int: *n* = 343 Ctr: *n* = 356	Alcohol use (12 months) Binge drinking (12 months) Drinking problems (12 months)	0.02 0.33 (0.17 to 0.47)* 00 (ns)
6. Adventure Trial^c^ [24, 63•]	AS, IMP, HOP, SS	HR secondary students Int: *n* = 558 Ctr: *n* = 437	Alcohol use (2 years) Drinking Q (2 years) Binge drinking (2 years) Binge drinking-freq (2 years) Binge drinking-growth (2 years) Drinking problems (2 years) Cannabis use (2 years)	0.68 (0.55 to 0.81)* 0.36 (0.23 to 0.49)* 0.88 (0.75 to 1.0)* 0.38 (0.25 to 0.50)* 2.07 (1.91 to 2.22)* 1.02 (0.88 to 1.16)* 0.06 (−0.06 to 0.18)^d^
7. Australian CAP^c^ Study [51]	AS, IMP, HOP, SS	HR secondary students Int: *n* = 202 Ctr: *n* = 291	Alcohol use (3 years) Binge drinking (3 years) Drinking problems (3 years)	0.47 (0.29 to 0.65)* 0.65 (0.46 to 0.84)* 0.54 (0.35 to 0.72)*
8. CBT for High AS [64]	AS	Community-recruited adults	Alcohol use Binge drinking Drinking problems (phys) Drinking problems (interper)	Not reported Not reported 0.64 0.48

All effect sizes are presented as *d* and calculated from standardized betas or from means and standard deviations as presented in published manuscripts. All effect sizes (*d*) were calculated using the procedure and effect size calculator described by Wilson (Practical Meta-Analysis Effect Size Calculator, David B. Wilson, Ph.D., George Mason University; http://www.campbellcollaboration.org/escalc/html/EffectSizeCalculator-SMD16.php)

*IMP* impulsivity, *SS* sensation seeking, *AS* anxiety sensitivity, *HOP* hopelessness, *Int* intervention, *Ctr* control, *QF* quantity and frequency, *Q* quantity, *CAP* Climate Schools and Preventure, *CBT* cognitive behavioural therapy, *Phys* physical, *interper* interpersonal

^a^Calculated based *p* = 0.06, as no other information was available, using the effect size calculator above

^b^Effect size (*d*) calculated on the basis of reported means and standard deviations

^c^Effect size (*d*) calculated on the basis of reported beta and standard error from latent growth models

^d^We report effect sizes for analyses in which those lost to follow-up are assigned substance use as these are the only results reported in Conrod’s study [62•] and allow for better comparisons across studies

What is striking about these results is the rather consistent moderate effects reported on most outcomes. For every study, Table 1 systematically reports on alcohol outcomes such as drinking (use or frequency), binge drinking (use or frequency) and alcohol problems (presence or severity). The average effect size across all studies and all outcomes is *d* = 0.47. For a small number of studies, one or two of these outcomes are not reported. Assuming that they are not reported because they did not yield significant effects, we assign a *d* = 0 to missing effect sizes and still yield an average effect size of *d* = 0.43 across all studies and measures (0.26 for alcohol use; 0.35 for binge drinking (including two unreported studies) and 0.46 for problem drinking). Three studies report illicit drug use outcomes, and the average of these reported outcomes is a *d* = 0.37.

Results across studies need to be interpreted in the context of possible limitations to generalizability. All studies relied on self-report to assess substance use outcomes, which might be susceptible to bias. However, all studies carefully describe a procedure for assuring confidentiality to participants and no consequences for reporting substance use. Some studies controlled for cluster-level effects (e.g. school), but not all. Only two studies included an active control intervention as a comparison group [22, 78]. None of these studies were able to mask intervention condition to participants or therapists but most described a procedure in which assessment of outcomes was conducted by experimenters blind to treatment condition.

Despite these methodological differences across studies, results indicate a consistent, moderate effect of brief, personality-targeted cognitive behavioural interventions on a number of substance use outcomes. They also indicate that the personality-targeted approach can be delivered in a variety of different settings and formats. There is also an emerging literature (not included in Table 1) showing that personality-targeted interventions also impact on mental health outcomes. Studies 3, 4, 5, 6 and 8 in Table 1 also report mental health outcomes in the following secondary analyses [44, 74, 78–80], and while there is some variability in results reported and measures used, significant effects are reported on depression, anxiety and conduct symptoms in four of five studies. Some additional secondary analyses report differential outcomes for personality subgroups or higher-risk individuals. Specifically, some researchers [24, 60, 61, 77, 81] report outcomes for specific personality risk groups, and findings are remarkably consistent across studies. SS youth appear at greater risk for early-onset drinking, binge drinking and cannabis use, and the intervention is particularly effective in delaying such use for these higher-risk youth; large effect sizes are reported. By contrast, the AS intervention was shown to be particularly effective in promoting abstinence from alcohol and alcohol-related problems, with AS-focused trials showing that alcohol outcomes are mediated by the effect of the AS on self-medication motives for drinking [44, 64].

### Conclusion

The personality-targeted approach has many advantages over more traditional CBT intervention approaches. First, in line with a more dimensional approach to understanding risk for psychopathology, interventions can be delivered following brief personality assessment, and their delivery is rarely delayed for the purpose of diagnostic clarity, which is often the case in clinical practice. Furthermore, because they target traits that are associated with risk for substance use, they can be helpful in the context of prevention but also appear to be equally helpful when targeting substance use problems that have already had their onset. Finally, the traits targeted in the personality-based approach are also relevant to non-addictive psychopathology and therefore have potential to be dually effective in reducing substance use and mental health concerns among individuals with concurrent disorders. Indeed, emerging results are showing that the personality-targeted approach holds promise for concurrent disorders [44, 64, 82].

Nevertheless, the research on personality-targeted interventions remains rather limited, relative to the potential applications of the approach. Despite studies showing that the SURPS personality dimensions are relevant to substance use in patients receiving treatment in general psychiatry, substance use or forensic settings [26, 32, 68, 83], personality-targeted interventions have yet to be tested in clinical settings with substance-dependent adults reporting concurrent mental health problems or with incarcerated individuals at risk of returning to substance use upon release from prison or following sentencing (e.g. for driving under the influence). Similarly, the SURPS dimensions have been shown to be highly relevant to smoking behaviour [30, 84], yet none of the published trials on personality-targeted interventions report smoking outcomes. The SURPS dimensions have also been shown to be implicated in other health behaviours, such as eating, drug injecting, sexual behaviours and risky driving (e.g. [85]), which suggests that the potential benefits of the personality-targeted approach are far reaching and might eventually provide solutions to a number of health problems facing society. Finally, the personality-targeted approach has been applied to the school setting for the purpose of preventing substance use in high-risk youth, but there are a number of other contexts that might prove to be opportunities for targeted prevention. For example, young people enlisted in the military, which has a long history of personality testing, might benefit from personality-targeted interventions prior to being exposed to service-related stressors known to increase risk for mental health problems and addiction. Similarly, pregnant women with a history of substance use or mental health concerns might also benefit from a prevention programme that focuses on their personality and coping strategies, rather than focusing directly on perinatal substance use, which remains a major taboo for pregnant women but continues to affect a significant number of newborns globally. The personality-targeted approach has a number of advantages over traditional substance use interventions because it does not necessarily refer to substance use and might therefore be less stigmatizing or threatening for such populations.

In conclusion, personality-targeted interventions for substance misuse have been applied and evaluated in a number of different contexts. A number of randomised trials indicate efficacy and effectiveness of a personality-targeted approach when applied to the school (secondary and undergraduate) and community context. Studies suggest, on average, moderate effect sizes on alcohol and illicit substance use outcomes, up to 3 years post-intervention. Studies also indicate promising results for non-addictive, mental health outcomes. More research is needed to understand the active ingredients of the personality-targeted approach, the most optimal delivery conditions and its potential to address other health behaviours.
